# Sleep in Elite Athletes and Nutritional Interventions to Enhance Sleep

**DOI:** 10.1007/s40279-014-0147-0

**Published:** 2014-05-03

**Authors:** Shona L. Halson

**Affiliations:** AIS Performance Recovery, Australian Institute of Sport, PO Box 176, Belconnen, ACT 2616 Australia

## Abstract

Sleep has numerous important physiological and cognitive functions that may be particularly important to elite athletes. Recent evidence, as well as anecdotal information, suggests that athletes may experience a reduced quality and/or quantity of sleep. Sleep deprivation can have significant effects on athletic performance, especially submaximal, prolonged exercise. Compromised sleep may also influence learning, memory, cognition, pain perception, immunity and inflammation. Furthermore, changes in glucose metabolism and neuroendocrine function as a result of chronic, partial sleep deprivation may result in alterations in carbohydrate metabolism, appetite, food intake and protein synthesis. These factors can ultimately have a negative influence on an athlete’s nutritional, metabolic and endocrine status and hence potentially reduce athletic performance. Research has identified a number of neurotransmitters associated with the sleep–wake cycle. These include serotonin, gamma-aminobutyric acid, orexin, melanin-concentrating hormone, cholinergic, galanin, noradrenaline, and histamine. Therefore, nutritional interventions that may act on these neurotransmitters in the brain may also influence sleep. Carbohydrate, tryptophan, valerian, melatonin and other nutritional interventions have been investigated as possible sleep inducers and represent promising potential interventions. In this review, the factors influencing sleep quality and quantity in athletic populations are examined and the potential impact of nutritional interventions is considered. While there is some research investigating the effects of nutritional interventions on sleep, future research may highlight the importance of nutritional and dietary interventions to enhance sleep.

## Background

Sleep has important biological functions regarding physiological processes, learning, memory, and cognition [1, 2]. This is evidenced by changes in almost all human physiological processes at the onset of sleep [3]. Although the function of sleep is not fully understood, it is generally accepted that it serves to permit recovery from previous wakefulness and/or prepare for functioning in the subsequent wake period. An individual’s recent sleep history therefore has a marked impact on their daytime functioning. Restricting sleep to less than 6 h per night for 4 or more consecutive nights has been shown to impair cognitive performance and mood [4], glucose metabolism [5], appetite regulation [6], and immune function [7]. This type of evidence has led to the recommendation that adults should obtain 8 h of sleep per night to prevent neurobehavioural deficits [8].

While there are considerable data available related to the amount of sleep obtained by adults in the general population, there are few published data related to the amount of sleep obtained by elite athletes. This appears to be a considerable oversight given that sleep has been recognized as an essential component of recovery from, and preparation for, high-intensity training [9–11]. At present there is almost no direct scientific evidence to support or refute the importance of sleep for athlete recovery. Therefore, if sleep debt is of significance to an athlete’s performance, there should be evidence that a decrease in athletic performance occurs as a consequence of sleep deprivation.

### Sleep Overview

#### Sleep Stages

**Fig. 1:**
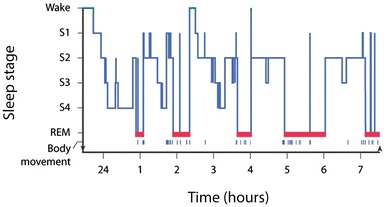
The progression of sleep stages across a single night in a normal young adult volunteer is illustrated in this sleep histogram. The text describes the ideal or average pattern. Reproduced from Carskadon and Dement [12], with permission. *REM* rapid eye movement, *S* stage

Sleep can be defined as a reversible behavioural state in which an individual is perceptually disengaged from and unresponsive to the environment [12]. Sleep is a complex physiological and behavioural state that has two primary states based on physiological parameters. These are rapid eye movement (REM) and non-rapid eye movement (NREM) sleep. An electroencephalogram in which electrodes measure brain electrical activity is used to identify the two states (Fig. 1). NREM sleep is divided into four stages, which are associated with a progressive increase in the depth of sleep [12]. REM sleep is characterized by muscle atonia, bursts of REM and dreaming. Therefore, REM sleep is considered to be a condition with an activated brain in a paralysed body.

It has been hypothesized that sleep, and in particular slow-wave sleep (SWS) or deep sleep, is important for recovery in athletes. While there is minimal research specifically in athletes, evidence in support of this theory includes the synchrony of growth hormone release with SWS in humans, the suggestion that optimum conditions for anabolism prevail during sleep, and studies showing the duration of SWS to be proportional to preceding wakefulness [13]. In addition, when SWS is decreased by deprivation, an increase in daytime sleepiness and a reduction in performance have been observed [14]. This is further support suggesting that SWS contributes to the recovery processes occurring during sleep.

Shapiro et al. [13] investigated sleep before and following a 92 km marathon in six subjects. The results indicated that total sleep time increased significantly over control times on each of the 4 nights after the marathon. Wakefulness was greatest on the night of the marathon, suggested to be related to muscle pain. The percentage of SWS increased on both nights 1 and 2. The quantitative increase in total sleep time, and particularly in SWS and the qualitative shift towards more stage 4 sleep immediately after metabolic stress, support the theory that sleep, and particularly SWS, is important for recovery in athletes.

#### Measuring Sleep

There are two commonly used methods to assess sleep. The first is polysomnography, which is a sleep study in which body functions such as brain activity (electroencephalogram), eye movements (electrooculogram), muscle activity (electromyogram), and cardiac activity (eletrocardiogram) are measured. Polysomnography provides information on sleep staging and is considered the ‘gold standard’ for assessing sleep quality and quantity. Information that can be obtained from polysomnography includes, but is not limited to, total sleep time, sleep-onset latency, wake after sleep onset, sleep efficiency, sleep fragmentation index, number of awakenings, time in each sleep stage, and sleep stage percentages. Polysomnography can be expensive, is labour intensive, and is often used primarily for assessing clinical sleep disorders.

A second method for measuring sleep is by means of actigraphy (wrist activity monitors), which are devices worn like a wristwatch that continuously record body movement (usually stored in 1-min epochs). Sleep diaries are also collected in which participants record the start and end date/time for all sleep periods (i.e. night-time sleeps and daytime naps). Data from sleep diaries and activity monitors are used to determine when participants are awake and when they are asleep. Essentially, all time is scored as awake unless the sleep diary indicates that the participant was lying down attempting to sleep, and the activity counts from the monitor are sufficiently low to indicate that the participant was immobile. When these two conditions are satisfied simultaneously, time is scored as sleep. Actigraphy is useful for understanding sleep patterns, and as it is non-invasive and is relatively easy to collect data over significant periods of time (~2 weeks of monitoring). Information that can be obtained from actigraphy includes total sleep time, sleep-onset latency, wake after sleep onset and sleep efficiency.

## Habitual Sleep Duration

According to a 2005 Gallup Poll in the US, the average self-reported sleep duration of healthy individuals is 6.8 h on weekdays and 7.4 h on weekends [15]. However, the sleep habits of elite athletes have only recently been investigated. Leeder et al. [16] compared the sleep habits of 26 elite athletes from olympic sports (canoeing, *n* = 11; diving, *n* = 14; rowing, *n* = 10; short-track speed skating, *n* = 11) using actigraphy over a 4-day period with that of age- and sex-matched non-sporting controls. The athlete group had a total time in bed of 8:36 ± 0:53 h:min, compared with 8:07 ± 0:20 in the control group. Despite the longer time in bed, the athlete group had a longer sleep latency (time taken to fall asleep) [18.2 ± 16.5 vs. 5.0 ± 2.5 min], and a lower sleep efficiency (estimate of sleep quality) than controls (80.6 ± 6.4 vs. 88.7 ± 3.6 %), resulting in a similar time asleep (6:55 ± 0:43 vs. 7:11 ± 0:25 h:min; mean ± SD). The results demonstrated that while athletes had a comparable quantity of sleep to controls, significant differences were observed in the quality of sleep [16].

While the above data were obtained during a period of normal training without competition, athletes may experience disturbed sleep before important competitions or games. Erlacher et al. [17] administered a questionnaire to 632 German athletes to assess possible sleep disturbances before competition. Of these athletes, 66 % (416) reported that they slept worse than normal at least once before an important competition. Of these 416 athletes, 70 % reported problems falling asleep, 43 % reported waking up early in the morning, and 32 % reported waking up at night. Factors such as thoughts about the competition (77 %), nervousness about the competition (60 %), unusual surroundings (29 %), and noise in the room (17 %) were identified as reasons for poor sleep [17].

In a study from the Australian Institute of Sport, athletes and coaches ranked sleep as the most prominent problem when they were asked about the causes of fatigue/tiredness [18]. Sleep characteristics ranked first when athletes were asked about the aspects of the clinical history that they thought were important. The study indicates that sleep complaints are common in athletes and highlights the need for sleep hygiene education.

Therefore, it appears that sleep disturbances in athletes can occur at two timepoints—before important competitions, and during normal training. This sleep disruption during normal training may be due to poor routine as a consequence of early training sessions, poor sleep habits (i.e. watching television in bed), nocturnal waking to use the bathroom, caffeine use, and excessive thinking/worrying/planning. While not documented in the literature, anecdotal evidence also suggests that athletes who compete at night also have significant difficulties falling asleep post-competition.

## Performance Effects of Sleep Deprivation and Extension

### Sleep Deprivation

A limited number of studies have examined the effects of sleep deprivation on athletic performance. Souissi et al. [19] measured cycling maximal power, peak power and mean power pre and post 24 and 36 h of sleep deprivation. Up to 24 h of waking, anaerobic power variables were not affected, but were impaired after 36 h without sleep [19]. Bulbulian et al. [20] examined knee extension and flexion peak torque before and after 30 h of sleep deprivation in trained men. Isokinetic performance decreased significantly following sleep deprivation. In support of the contention that the effects of sleep deprivation are task specific, 64 h of sleep deprivation significantly reduced vertical jump performance and isokinetic knee extension strength, while isometric strength and 40 m sprint performance were unaffected [21].

Blumert et al. [22] examined the effects of 24 h of sleep deprivation in nine US college-level weightlifters in a randomized counterbalanced design. There were no differences in any of the performance tasks (snatch, clean and jerk, front squat, and total volume load and training intensity) following 24 h of sleep deprivation when compared with no sleep deprivation [22]. However, mood state, as assessed by the profile of mood states, was significantly altered, with confusion, vigour, fatigue and total mood disturbance all negatively affected by sleep deprivation.

Reductions in endurance running performance have been observed following 24 h of sleep deprivation [23]. Eleven men performed a 30-min preload treadmill run at 60 % maximal oxygen uptake followed by a 30-min self-paced distance test following a normal night’s sleep (496 ± 18 min) and a night without sleep (0 min). Following the night of sleep deprivation, less distance was covered during the distance test (6,037 ± 757 m) when compared with control (6,224 ± 818 m; mean ± SD). The authors suggested that in the absence of significant alterations in physiological parameters and pacing, the reduced performance was likely to be a result of an increased perception of effort.

Skein et al. [24] reported significant decreases in mean and total sprint time following 30 h of sleep deprivation in ten male team-sport athletes. In addition, sleep deprivation resulted in altered repeat sprint pacing strategies, reduced muscle glycogen content, reduced peak voluntary force, reduced voluntary activation, and increased perceptual strain [24]. The authors suggested that altered afferent feedback from reduced muscle glycogen content and/or increased perceptual strain may have reduced muscle recruitment and thereby reduced repeated sprint performance.

While the above studies provide some insight into the relationship between sleep deprivation and performance, most athletes are more likely to experience acute bouts of partial sleep deprivation in which sleep is reduced for several hours on consecutive nights [25].

### Partial Sleep Deprivation

A small number of studies have examined the effect of partial sleep deprivation on athletic performance. Reilly and Deykin [25] reported decrements in a range of psychomotor functions after only 1 night of restricted sleep; however, gross motor function such as muscle strength, lung power, and endurance running were unaffected. Reilly and Hales [26] reported similar effects in women following partial sleep deprivation, with gross motor functions being less affected by sleep loss than tasks requiring fast reaction times.

The effect of 2.5 h of sleep deprivation per night over 4 nights was measured in eight swimmers [27]. No effect of sleep loss was observed when investigating back and grip strength, lung function, or swimming performance. However, mood state was significantly altered with increases in depression, tension, confusion, fatigue, and anger, and decreases in vigour. Reilly and Piercy [28] found a significant effect of sleep loss on maximal bench press, leg press, and dead lifts, but not maximal bicep curl. Submaximal performance was, however, significantly affected on all four tasks and to a greater degree than maximal efforts. The greatest impairments were found later in the protocol, suggesting a cumulative effect of fatigue from sleep loss [28].

From the available research it appears that submaximal prolonged tasks may be more affected than maximal efforts, particularly after the first 2 nights of partial sleep deprivation [28]. While partial sleep deprivation may be common in the elite athlete, much of the research in this area has not examined the elite athlete, and the degree of sleep deprivation in those studies may be more than experienced during normal training phases. In addition, understanding the sleep deprivation that may occur during competition phases and how this affects performance is an important area of future research.

### Effects of Sleep Extension

Another means of examining the effect of sleep on performance is to extend the amount of sleep an athlete receives and determine the effects on subsequent performance. Mah et al. [29] instructed six basketball players to obtain as much extra sleep as possible following 2 weeks of normal sleep habits. Faster sprint times and increased free-throw accuracy were observed at the end of the sleep extension period. Mood was also significantly improved, with increased vigour and decreased fatigue [29]. The same research group also increased the sleep time of swimmers from their usual sleep amount to 10 h per night for 6–7 weeks. Following this period, 15 m sprint, reaction time, turn time, and mood all improved [30].

### Effects of Napping

Athletes suffering from some degree of sleep loss may benefit from a brief nap, particularly if a training session is to be completed in the afternoon or evening. Waterhouse et al. [31] investigated the effects of a lunchtime nap on sprint performance following partial sleep deprivation (4 h of sleep). Following a 30-min nap, 20 m sprint performance was increased, alertness was increased, and sleepiness was decreased when compared with the no-nap trial. In terms of cognitive performance, sleep supplementation in the form of napping has been shown to have a positive influence on cognitive tasks following a night of sleep deprivation (2 h) [32]. Naps can markedly reduce sleepiness and can be beneficial when learning skills, strategy or tactics [32]. Napping may also be beneficial for athletes who have to wake early routinely for training or competition and those who are experiencing sleep deprivation [32].

The data from these small number of studies on sleep extension and napping suggest that increasing the amount of sleep an athlete receives may significantly enhance performance. Given that recent data suggest that elite athletes have a lower total sleep time and lower sleep efficiency than non-athletes [16], it appears logical that increasing the quantity of sleep obtained may result in an increase in athletic performance.

### Other Consequences of Sleep Deprivation

There are numerous other consequences of sleep deprivation, in addition to reduced exercise performance, that have been investigated in the general population. While those studies have not focused on athletes, some of the changes that occur in cognition, pain perception, immunity and inflammation, and metabolism and endocrine function may be relevant to the elite athlete. While it is beyond the scope of this review to examine research in each of these areas comprehensively, a summary is provided below and reference made to review articles where appropriate.

#### Cognition

Sleep research has identified correlations between various sleep parameters (sleep architecture and efficiency) and attention, concentration, skills, perceptual function, language, memory, and executive and intellectual function [33]. One of the most studied areas of sleep deprivation research relates to effects on alertness and performance [34]. In this instance ‘performance’ is generally defined as goal-directed behaviour requiring mental effort. Performance deficits due to sleep deprivation are well acknowledged and understood, and it is estimated that the consequences cost billions of dollars worldwide per year due to accidents, direct healthcare costs, and reduced efficiency and productivity [34]. Learning and memory deficits are also evident after sleep deprivation. It appears that sleep is important not only following learning for consolidation of memory, but also for preparing the brain for next-day memory formation [35].

Adequate cognitive function plays a key role in many sports, especially team sports. Likewise, the ability to consolidate skill memory is important for skill performance, and thus sleep deprivation may have a negative impact on performance by reducing cognitive function. While there is an absence of scientific data to date examining the role of sleep deprivation on the risk of acute injury due to decreased concentration, poor execution, or reduced reaction times, it is possible that sleep deprivation may result in an increased predisposition to injury.

#### Pain Perception

It is well accepted that individuals with chronic pain frequently report disturbed sleep (changes in continuity of sleep as well as sleep architecture). However, there is also recent evidence suggesting that sleep deprivation may cause or modulate acute and chronic pain [36]. Sleep deprivation may thus enhance or cause pain, and pain may disturb sleep by inducing arousals during sleep. A cycle may then eventuate, starting with either pain or sleep deprivation, with these two issues maintaining or augmenting each other [36].

Athletes may experience pain as a result of training, competition and/or injury. Evidence, although minimal at this stage, suggests that athletes may also have lower sleep quality and quantity than the general population [16]. Therefore, appropriate pain management as well as adequate sleep is likely to be very important for athletes from both a pain and sleep perspective.

#### Immunity and Inflammation

Similar to the evidence regarding sleep and pain, a bidirectional relationship has also been proposed regarding sleep and immunity. Increasing amounts of evidence suggest that sleep deprivation can have detrimental effects on immune function, and that immune responses feed back on sleep architecture [37]. In a recent review examining the link between sleep and immunity, it was concluded that sleep improves immune responses and that most immune cells have their peak pro-inflammatory activity at night [37]. Disruptions in endocrine and physiological circadian rhythms due to sleep deprivation may result in impaired immune responses, giving rise to an increased risk of illness.

A recent study examined immune function in participants who naturally slept for short (<7 h), normal (7–9 h) or long (>9 h) durations on the night before testing [38]. Short sleep duration was associated with 49 % higher T-cell function in response to an antigen and 30 % lower natural killer cell activity when compared with normal sleep. While the implication of high T-cell activity is unclear, it was suggested that this may be related to autoimmune diseases or low-grade systemic inflammation [38].

Increasing sleep duration (from 8 to 10 h) or napping (30 min) following 1 night of sleep deprivation (only 2 h of sleep) both resulted in a return of leukocyte values to normal ranges [39]. Saliva cortisol has also been shown to decrease immediately after a nap [39].

Markers of the acute inflammatory system, such as interleukin (IL)-1β, tumour necrosis factor-α, IL-6, and C-reactive protein are all influenced following the manipulation of sleep [40]. In addition, patients with insomnia and sleep apnoea have elevated inflammatory markers [40]. This increased systemic inflammation may play an important role in homeostatic functions such as insulin sensitivity and metabolism, blood pressure and, of course, sleep itself.

From the current evidence indicating that very high levels of physical activity may have implications for the immune system [41], short amounts of sleep may reduce immune function, and extended sleep or napping may improve immune function, it appears that poor sleep and the resultant decreased immunity may have a negative influence on athletic performance.

#### Metabolism and Endocrine Function

**Fig. 2:**
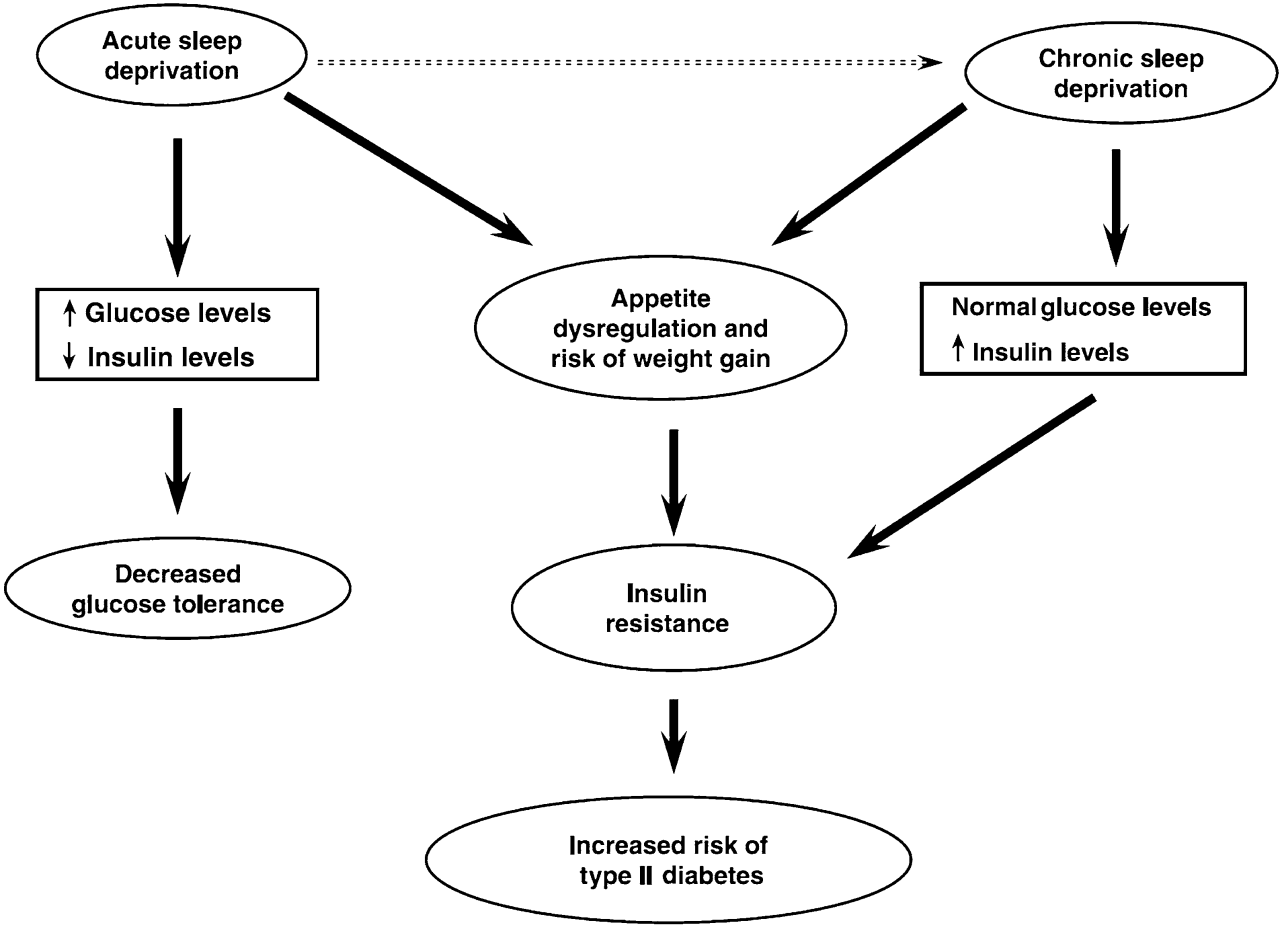
Schematic diagram of the potential pathways leading from sleep loss to diabetes risk. Reproduced from Spiegel et al. [43], with permission

Both laboratory and epidemiological studies support the notion that chronic partial sleep loss can increase the risk of obesity and diabetes [42]. Potential mechanisms include changes in glucose regulation by insulin resistance, dysregulation of neuroendocrine control of appetite and/or increased energy intake [42, 43] (Fig. 2).

The exact mechanism by which decreased sleep influences glucose metabolism is thought to be multifactorial and includes decreased brain glucose utilization, alterations in sympathovagal balance, increased evening cortisol, extended night-time growth hormone secretion, and pro-inflammatory processes [44].

Leptin and ghrelin are hormonal regulators of food intake, with leptin exerting inhibitory effects on food intake and ghrelin being an appetite-stimulating hormone [44]. Several studies have shown that sleep deprivation results in decreases in leptin and increases in ghrelin [44]. Sleep restriction has also been shown to increase hunger and appetite, especially relating to carbohydrate-rich foods [6].

In addition to alterations in appetite-regulating hormones, the function of two major neuroendocrine axes are also negatively affected (hypothalamic-pituitary-adrenal axis and hypothalamic-pituitary-gonadal axis) [45]. This results in both increases in the secretion of catabolic hormones such as cortisol, and changes in the secretion of anabolic hormones such as testosterone and insulin-like growth factor 1. It has been proposed that these changes in hormone patterns may reduce protein synthesis and/or increase proteolysis, thereby impairing muscle recovery [45].

When transitioning from wakefulness to sleep, there is a shift of autonomic balance to that of parasympathetic dominance [46]. Therefore, sleep is associated with a decrease in both sympathetic activity and catecholamine levels, and sleep loss is associated with an increase in these variables [46]. Sleep deprivation has also been shown to have a negative influence on the responsiveness of adrenocorticotropic hormone, adrenaline, noradrenaline, and serotonin (5-HT) receptor sensitivity [46]. Over time, this may lead to altered stress system responsiveness, similar to that seen in mood disorders.

Chronic partial sleep deprivation in athletes may result in altered glucose metabolism and neuroendocrine function, causing concern regarding carbohydrate metabolism, appetite, food intake, and protein synthesis. These factors can all influence an athlete’s nutritional, metabolic, and endocrine status negatively and hence potentially reduce athletic performance.

## Nutritional Interventions that may Influence Sleep

### Background

Research has identified a number of neurotransmitters associated with the sleep–wake cycle. These include 5-HT, gamma-aminobutyric acid (GABA), orexin, melanin-concentrating hormone, cholinergic, galanin, noradrenaline, and histamine [47]. Therefore, nutritional interventions that act on these brain neurotransmitters may also influence sleep.

**Fig. 3:**
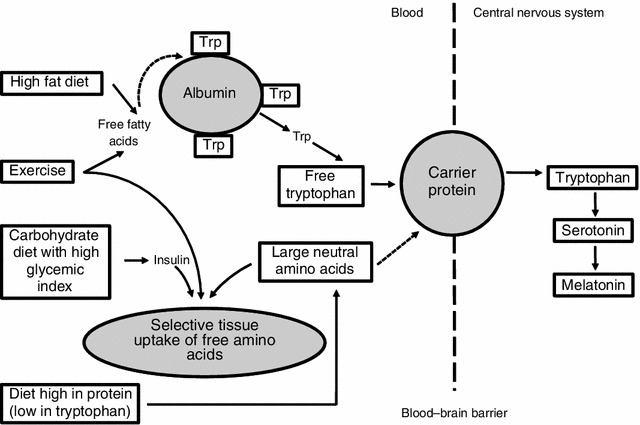
Effects of diet on tryptophan (Trp) uptake and the central nervous system. Adapted from Grimmett and Sillence [49], with permission. *Dashed line* indicates blood–brain barrier

Dietary precursors can influence the rate of synthesis and function of a small number of neurotransmitters, including 5-HT [48]. Figure 3 depicts the means by which diet may influence the central nervous system through the production of 5-HT and melatonin [49]. Synthesis of 5-HT is dependent on its precursor availability in the brain, the amino acid l-tryptophan (Trp). Trp is transported across the blood–brain barrier by a system that shares other transporters including a number of large neutral amino acids (LNAA). Thus, the ratio of Trp/LNAA in the blood is crucial to the transport of Trp into the brain.

Ingestion of other forms of protein generally decrease the uptake of Trp into the brain, as Trp is the least abundant amino acid and therefore other LNAA are preferentially transported into the brain [48]. Carbohydrate, however, increases brain Trp by insulin stimulation of LNAA into skeletal muscle, which results in an increase in free Trp [50].

Melatonin is a hormone released from the pineal gland that transmits information regarding the light–dark cycle, and retinal light exposure results in a suppression of melatonin [51]. Melatonin can influence the sleep–wake cycle, by a sleep-promoting effect, and therefore a number of nutritional interventions aim to increase melatonin by the manipulation of Trp.

In summary, Fig. 3 describes the means by which melatonin production may be increased by either increasing Trp intake or reducing the relative plasma concentration of LNAA. This can be achieved by several means, including a high protein diet that contains more tryptophan than LNAA; ingestion of carbohydrate, which may increase the ratio of free Trp to branched-chain amino acids and facilitate the release of insulin, which promotes the uptake of branched-chain amino acids into the muscle; ingestion of a high-fat meal, which may increase free fatty acids and result in increased free Trp; and exercise, which can influence both free fatty acids and insulin.

### Carbohydrate

A small number of studies have investigated the effects of carbohydrate ingestion on indices of sleep quality and quantity. Porter and Horne [52] provided six male subjects with a high-carbohydrate meal (130 g), a low-carbohydrate meal (47 g), or a meal containing no carbohydrate, 45 min before bedtime. The high-carbohydrate meal resulted in increased REM sleep, decreased light sleep, and wakefulness [52]. However, the caloric content of the meals was not matched in the study.

The effect of meal versus drink (with high-, normal-, and low-carbohydrate content) versus water at various time intervals before sleep has also been studied [53]. Solid meals enhanced sleep-onset latency (time taken to fall asleep) up to 3 h after ingestion, and the liquid meal was slightly better than water. There was no effect of meal or drink composition on sleep.

Afaghi and colleagues [54, 55] conducted two studies investigating carbohydrate ingestion before sleep in healthy men. In the first study, high or low glycemic index (GI) meals were given 4 h or 1 h before sleep [54]. The high GI meal significantly improved sleep-onset latency above the low GI meal, and providing the meal 4 h before sleep was better than providing the meal 1 h before sleep. In the second study, the authors compared a very low carbohydrate diet (1 % carbohydrate, 61 % fat, 38 % protein) with a control diet (72 % carbohydrate, 12.5 % fat, 15.5 % protein) matched for kilojoule content, 4 h before sleep [55]. The very-low-carbohydrate diet increased SWS (deep sleep) and all stages of NREM, whereas the control diet decreased REM. Finally, Jalilolghadr et al. [56] provided eight children with either a high GI (200 mL milk plus glucose) or low GI drink (200 mL milk plus honey) 1 h before bedtime. In that study, the high GI drink increased arousals to a greater extent than the low GI drink.

From the limited and somewhat contradictory nature of the above studies, it appears that high GI foods may be beneficial if consumed more than 1 h before bedtime and that solid meals may be better than liquid meals.

### Mixed Composition Meals

Only a small number of studies have investigated the effects of meals of varying composition or drinks on sleep. Hartmann et al. [57] provided a drink with the evening meal, which was either high fat (90 g), high carbohydrate (223 g), or high protein (30 g). Results showed no effect of any of the drinks on sleep. Zammit et al. [58] examined the effect of a liquid meal high (994 kcal) or low (306 kcal) in caloric content provided at lunch, compared with no meal, on daytime naps. Both liquid meals demonstrated increased sleep time in stages 2 and 3 when compared with no meal; however, there were no differences in sleep-onset latency [58].

Again, there is very limited research in this area; however, it appears that reduced caloric intake may result in poor sleep.

### Diet

The above-mentioned studies have examined acute nutritional manipulations on sleep. Research has also been conducted that investigates more chronic manipulations of habitual dietary intake. Kwan et al. [59] provided six healthy women with a low-carbohydrate (50 g/day) diet for 7 days, and reported increased REM latency when compared with sleep before the intervention when subjects were consuming their usual diet. Lacey et al. [60] also studied women for 7 days with either high protein (>100 g), low protein (<15 g), or normal daily protein intakes. High-protein intakes resulted in increased restlessness, while low-protein intakes resulted in lower amounts of SWS, but there were no differences in total sleep time. While it is difficult to draw definitive conclusions from the study, it appears that altering protein intake can influence aspects of sleep architecture.

In a recent comprehensive study, Lindseth et al. [61] manipulated the diet of 44 adults for 4 days. Diets were either high protein (56 % protein, 22 % carbohydrate, 22 % fat), high carbohydrate (22 % protein, 56 % carbohydrate, 22 % fat), or high fat (22 % protein, 22 % carbohydrate, 56 % fat). Diets higher in carbohydrate resulted in shorter sleep-onset latencies, and diets higher in protein resulted in fewer wake episodes [61]. Finally, Grandner et al. [62] examined the dietary intake (by means of questionnaires) of 459 postmenopausal women over 7 days. The only significant finding was that fat intake was negatively associated with total sleep time.

From the above studies it appears that diets high in carbohydrate may result in shorter sleep latencies, diets high in protein may result in improved sleep quality, and diets high in fat may negatively influence total sleep time.

### Tryptophan

As mentioned above, the synthesis of 5-HT is dependent on the availability of its brain precursor, Trp. Furthermore, 5-HT is a precursor to melatonin in the pineal gland. There have been numerous investigations of the effects of tryptophan supplementation on sleep (for review see Silber and Schmitt [48]), and it appears that Trp doses as low as 1 g can improve sleep latency and subjective sleep quality.

### Melatonin

Research investigating the use of melatonin for primary insomnia demonstrates inconclusive results [63]. A meta-analysis reported a reduction in sleep-onset latency of 7.2 min, and concluded that while melatonin appeared safe for short-term use, there was no evidence that melatonin was effective for most primary sleep disorders [64].

Another recently investigated intervention is tart cherry juice. The ingestion of tart cherries may increase exogenous melatonin, and when consumed over a 2-week period was shown to improve subjective insomnia symptoms when compared with placebo [65]. It has also been shown to result in modest improvements in sleep time and quality [66]. Tart cherries also contain antioxidant and anti-inflammatory phytochemicals that may influence sleep by means of cytokines associated with the sleep–wake cycle [66]; therefore, it may not be melatonin availability per se that results in reductions in sleep complaints. Furthermore, melatonin in supplement or pharmacological form may have side effects such as headaches, nausea, daytime sleepiness, and vivid dreams and nightmares [63].

### Valerian

Valerian is a herb that binds to GABA type A receptors and is thought to induce a calming effect [67] by regulation of excitability of the nervous system. Results of a meta-analysis investigating the efficacy of valerian showed a subjective improvement in sleep quality [68], although improvements in quantitative measures of sleep have not been demonstrated [63]. While valerian is one of the more common ingredients found in supplements claiming to promote sleep, side effects such as drowsiness, dizziness and allergic reactions can be observed [63]. Furthermore, GABA may influence growth hormone secretion, which may have potential implications for athletes and anti-doping regulations [69].

### Other Nutritional Interventions

Nucleotides are thought to be involved in the physiological function of sleep, in particular uridine monophosphate (5′UMP) and adenosine monophosphate (5′AMP). 5′UMP demonstrates a depressive effect on the central nervous system, and one study that administered low doses before sleep showed improvements in some sleep indices [70]. 5′AMP has hypnotic properties, and levels of this nucleotide decline during wakefulness [71] and act on adenosine A_2A_ receptors in ventrolateral nuclei [72]. These nucleotides have been studied by investigations of the hypnotic effects of infant formula [71]. In that study the sleep-promoting formula contained high levels of tryptophan and carbohydrate, low levels of protein, 5′UMP and 5′AMP. Fifty-four children were monitored over 1 week using actigraphy, with results showing increased time in bed and increased sleep efficiency. The authors suggest that these results support the concept of chrononutrition, that is, the time of day at which food is ingested affects different biological rhythms such as sleep and wakefulness. However, no blood measures were made and thus it is not possible to determine whether the compounds were transported from the digestive system to the bloodstream.

Glycine (a non-essential amino acid) functions as an inhibitory neurotransmitter in the central nervous system and also acts as a co-agonist of glutamate receptors. In a Japanese study [73], glycine has been shown to improve subjective sleep. Yamadera et al. [74] also reported shorter sleep-onset latencies measured by polysomnography. The authors suggested that potential mechanisms involve increased vasodilation and thus lowering of core temperature and increased extracellular 5-HT release in the prefrontal cortex of rats [74].

l-Theanine is an amino acid analogue present in tea but not coffee, and demonstrates pharmacological actions such as promoting feelings of calmness and decreasing alertness. Jang et al. [75] reported that l-theanine partly counteracted the caffeine-induced decrease in SWS in rats.

There are also numerous other traditional sleep aids, including passionflower, kava, St. John’s wort, lysine, magnesium, lavender, skullcap, lemon balm, magnolia bark, 5-hydroxytryptamine, and GABA. While the majority of these forms of intervention have not been adequately investigated in the scientific literature, many can be found in sleep aid supplements that can be purchased over the counter or at health food suppliers.

## Practical Applications

In the first instance, athletes should focus on utilizing good sleep hygiene to maximize sleep quality and quantity. While research is minimal and somewhat inconclusive, several practical recommendations may be suggested:

- High GI foods such as white rice, pasta, bread, and potatoes may promote sleep; however, they should be consumed more than 1 h before bedtime.
- Diets high in carbohydrate may result in shorter sleep latencies.
- Diets high in protein may result in improved sleep quality.
- Diets high in fat may negatively influence total sleep time.
- When total caloric intake is decreased, sleep quality may be disturbed.
- Small doses of tryptophan (1 g) may improve both sleep latency and sleep quality. This can be achieved by consuming approximately 300 g of turkey or approximately 200 g of pumpkin seeds.
- The hormone melatonin and foods that have a high melatonin concentration may decrease sleep onset time.
- Subjective sleep quality may be improved with the ingestion of the herb valerian; however, as with all supplements, athletes should be aware of potential contaminants as well as the inadvertent risk of a positive drug test.

## Conclusion

Sleep deprivation can have significant effects on athletic performance, especially submaximal, prolonged exercise. From the limited evidence, it appears that athletes may be obtaining less than 8 h of sleep per night. Increasing sleep (sleep extension) or napping may be useful to increase the total number of hours of sleep.

Changes in glucose metabolism and neuroendocrine function as a result of chronic, partial sleep deprivation may result in alterations in carbohydrate metabolism, appetite, food intake and protein synthesis. These factors may negatively influence an athlete’s nutritional, metabolic and endocrine status, and hence potentially reduce athletic performance. While there is some research investigating the effects of nutritional interventions on sleep, future research may highlight the importance of nutritional and dietary interventions to enhance sleep.
